# Effects of household access to water, sanitation, and hygiene services on under-five mortality in Sub-Saharan Africa

**DOI:** 10.3389/fpubh.2023.1136299

**Published:** 2023-04-27

**Authors:** Nicolas Gaffan, Alphonse Kpozehouen, Cyriaque Degbey, Yolaine Glele Ahanhanzo, Moussiliou Noël Paraïso

**Affiliations:** ^1^Department of Epidemiology and Biostatistics, Regional Institute of Public Health, University of Abomey-Calavi, Ouidah, Benin; ^2^Department of Environmental Health, Regional Institute of Public Health, University of Abomey Calavi, Ouidah, Benin; ^3^University Hospital Hygiene Clinic, National Hospital and University Centre Hubert Koutoukou Maga, Cotonou, Benin; ^4^Department of Health Promotion, Regional Institute of Public Health, University of Abomey-Calavi, Ouidah, Benin

**Keywords:** water, sanitation, hygiene, demographic and health survey, child, under-five mortality, household, Sub-Saharan Africa

## Abstract

**Introduction:**

Sub-Saharan Africa has the highest under-five mortality rate and is among the regions where people have the least access to adequate Water, Sanitation, and Hygiene (WASH) services. The work aimed to investigate the effects of WASH conditions faced by children on under-five mortality in Sub-Saharan Africa.

**Methods:**

We carried out secondary analyses using the Demographic and Health Survey datasets of 30 countries in Sub-Saharan Africa. The study population consisted of children born within 5 years preceding the selected surveys. The dependent variable was the child’s status (1 = deceased versus 0 = alive) on the survey day. The individual WASH conditions in which children live were assessed in their immediate environment, i.e., at the level of their households of residence. The other explanatory variables were related to the child, mother, household, and environment. Following a description of the study variables, we identified the predictors of under-five mortality using a mixed logistic regression.

**Results:**

The analyses involved 303,985 children. Overall, 6.36% (95% CI = 6.24–6.49) of children died before their fifth birthday. The percentage of children living in households with access to individual basic WASH services was 58.15% (95% CI = 57.51–58.78), 28.18% (95% CI = 27.74–28.63), and 17.06% (95% CI = 16.71–17.41), respectively. Children living in households using unimproved water facilities (aOR = 1.10; 95% CI = 1.04–1.16) or surface water (aOR = 1.11; 95% CI = 1.03–1.20) were more likely to die before five than those coming from households with basic water facilities. The risk of under-five mortality was 11% higher for children living in households with unimproved sanitation facilities (aOR = 1.11; 95% CI = 1.04–1.18) than for those with basic sanitation services. We found no evidence to support a relationship between household access to hygiene services and under-five mortality.

**Conclusion:**

Interventions to reduce under-five mortality should focus on strengthening access to basic water and sanitation services. Further studies are needed to investigate the contribution of access to basic hygiene services on under-five mortality.

## 1. Introduction

With the adoption and commitment of the United Nations member states to the Millennium Development Goals (MDGs) and then the Sustainable Development Goals (SDGs), the progress already observed since the 1990s in child health has been maintained and led, among other things, to a significant decrease in global child mortality. The worldwide under-five mortality rate decreased by half during 2000–2020, from 76 to 37 deaths per 1,000 live births (1). Despite this progress, much remains in child survival and well-being. Indeed, between 2016 and 2030, 56 to 94 million children are projected to die by 5 years (2). In 2020 alone, 4.8 to 5.5 million children died before their fifth birthday (1). The spatial distribution of these fatalities shows that children have unequal risks of leading a healthy life, depending on where they are born or live (1). Sub-Saharan Africa (SSA) was home to 54% (2.7 million) of all under-five fatalities in 2020 while accounting for 27% of live births (1). Besides, the under-five mortality rate was 74 deaths per 1,000 live births, twice the global figure and about 15 times higher than in Europe and North America (1).

In the literature, we find a range of studies on the predictors of under-five mortality, particularly in SSA. These works have generated the evidence necessary to design and implement appropriate strategies. Overall, they have highlighted the effects of child, maternal, household, and environmental factors. Female children have a lower risk of under-five mortality than their male counterparts (3–10). Twins have a higher under-five mortality rate than singles (3, 4, 10, 11). Other significant factors include a short inter-genital interval and low birth weight (7, 9–11). Indoor air pollution related to smoking or cooking fuels also appears to be associated with under-five mortality (6, 10). Children with higher-educated mothers have a higher probability of survival than those with low or uneducated mothers (5–7, 10–12). There is also a significant inverse relationship between wealth index (at household and national levels) and under-five mortality, as well as regional disparities, notably against rural areas (3–6, 9, 11–14).

In the present study, we hypothesize that in SSA, Water, Sanitation, and Hygiene (WASH) conditions in children’s immediate environment significantly affect under-five mortality. It is clear that SSA has the highest under-five mortality rate and is among the regions where people have the least access to adequate WASH services (1, 15). By 2020, 65, 33, and 26% of Africans had access to at least individual basic WASH services, respectively, compared to 90, 78, and 71% globally (15). In North America and Europe, access to basic water and sanitation services is almost universal (15). The prevalence of surface water consumption and open defecation in SSA was 7 and 18%, respectively, compared to 2 and 6% globally (15). The literature suggests that the association between WASH conditions and under-five mortality remains poorly documented. A systematic review in 2018 provided very low-quality evidence for a reduction in mortality (<18 years) (16). However, recent meta-analyses have found a positive and significant relationship between WASH conditions to which children under five are exposed and diarrheal diseases, which are among the leading causes of death (17–19). A multicenter study in 2021 found that the risk of under-five mortality was 11% higher among children living in households with access to unimproved water sources (9). Additionally, a 2020 study in low and middle-income countries found a 9–12% higher relative risk of under-five mortality among children living in households without adequate sanitation (14). However, in the latter two studies, the authors did not explore the hygiene component (9, 14). The current work aimed to study the effects of WASH conditions faced by children on under-five mortality in SSA.

## 2. Methods

### 2.1. Study setting

SSA is the area of Africa located south of the Sahara and divided into four subregions and 48 countries, namely Central Africa (Angola, Cameroon, Central African Republic, Chad, Equatorial Guinea, Gabon, Republic of Congo, Democratic Republic of the Congo, Sao Tome and Principe), West Africa (Benin, Burkina Faso, Cape Verde, Ivory Coast, Gambia, Ghana, Guinea, Guinea-Bissau, Liberia, Mali, Mauritania, Niger, Nigeria, Senegal, Sierra Leone, and Togo), East Africa (Burundi, Comoros, Djibouti, Ethiopia, Eritrea, Kenya, Mauritius, Madagascar, Malawi, Mozambique, Rwanda, Tanzania, Somalia, South Sudan, Uganda, Zambia, and Zimbabwe), and Southern Africa (Lesotho, Namibia, South Africa, Botswana, and Eswatini). Together, they cover 24.3 million km^2^. SSA has the fastest population growth in the world since 2000 (around 2.7% per year compared to 1.1 globally during 2015–2020), a young population (median age of 18.2 years compared to 29.6 globally, and 43% under 15 compared to 26% globally in 2015), high fertility (4.8 children per woman compared to 2.5 globally during 2015–2020), and the highest mortality (61 years of life expectancy compared to 72 years globally during 2015–2020) (20). Economically and socially, the region remains the most disadvantaged (20).

### 2.2. Study type and data sources

The study consisted of secondary analyses based on Demographic and Health Survey (DHS) datasets of countries in SSA. The DHS are national surveys conducted since 1984 to produce indicators on, among other things, the demographic and health situation of women aged 15–49 and children under five (21). To date, there are more than 300 DHS conducted in more than 90 countries through eight 5-year phases: Phase 1 (1984–1990), Phase 2 (1989–1993), Phase 3 (1992–1998), Phase 4 (1997–2003), Phase 5 (2003–2008), Phase 6 (2008–2013), Phase 7 (2013–2018), Phase 8 (2018–2023) (21).

### 2.3. Study population

The study population included children born less than 5 years before the most recent DHS from 2010 to 2021 in SSA, for which recode datasets were available by 31 August 2022. Furthermore, only the “first twin” was included for children born from multiple pregnancies. The study did not cover children who were not usually residents in the surveyed households or those with missing data for the variables of interest.

**Figure 1:**
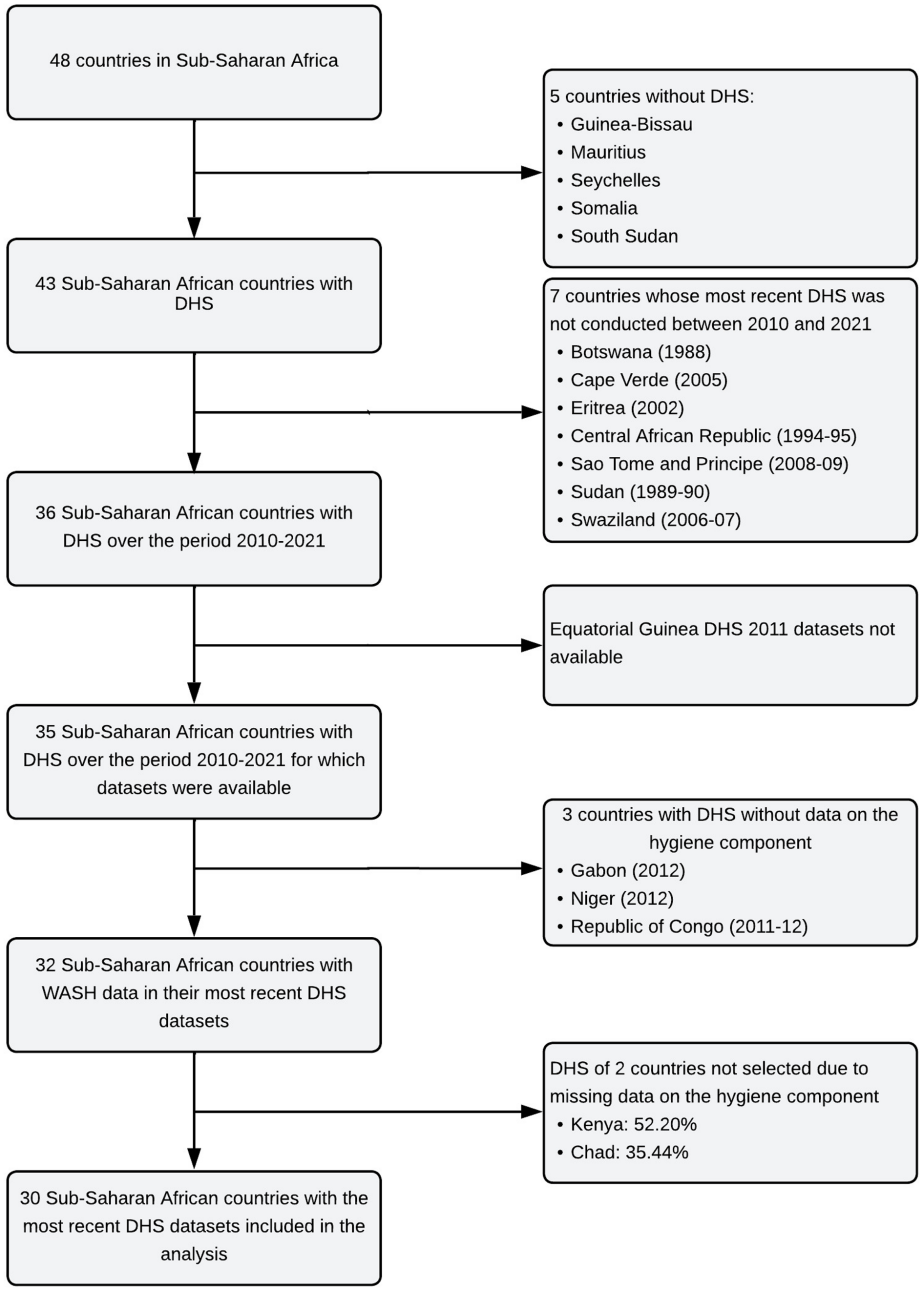
Flowchart for the selection of national DHS.

Of the 48 countries in SSA, 43 had already conducted DHS, and 36 from 2010 to 2021. Seven countries did the most recent DHS before the period of interest: Botswana (1988), Cape Verde (2005), Eritrea (2002), Central African Republic (1994–95), Sao Tome and Principe (2008–09), Sudan (1989–90) and Swaziland (2006–07). Data from the 2011 DHS in Equatorial Guinea were not available for download. The household datasets (HR) in Gabon (2012), Niger (2012), and the Republic of Congo (2011–12) did not include data on the hygiene component and were excluded. There were 52.20 and 35.44% missing data for the variable on hygiene in Kenya and Chad, respectively. These two countries were not included. Ultimately, the data analysis covered 30 countries (Figure 1): Angola (2015–16), Benin (2017–18), Burkina-Faso (2010), Burundi (2017–18), Cameroon (2018), Comoros (2012), Democratic Republic of Congo (2013–14), Ivory Coast (2011–12), Ethiopia (2016), Gambia (2019–20), Ghana (2014), Guinea (2018), Lesotho (2014), Liberia (2019–20), Madagascar (2021), Malawi (2015–16), Mali (2018), Mauritania (2019–21), Mozambique (2011), Namibia (2013), Nigeria (2018), Uganda (2016), Rwanda (2019–20), Senegal (2019), Sierra Leone (2019), South Africa (2016), Tanzania (2015–16), Togo (2013–14), Zambia (2018), and Zimbabwe (2018). Data on children were extracted, cleaned, harmonized, and merged for these countries.

Most of the DHS in the selected countries used a two-stage stratified sampling technique. First, the first level of administrative division of national territories are stratified into urban and rural areas. Then in each stratum, a specific number of Primary Sample Units (PSUs) are drawn in the first stage using a systematic probability proportional to size. Usually, this happens by using the list of enumeration areas established during the most recent population census surveys. In the second step, after listing the households within the selected PSUs, a given number of households were selected by systematic sampling with equal probability. Details and specifics of the sampling process for the different targets, including children under five, are available in the final reports of the selected country surveys published on “www.dhsprogram.com.” In the end, the analyses involved 303,985 participants. Table 1 highlights the distribution of surveyed children by country.

**Table 1:** Distribution of surveyed children by country.

Subregions	Countries	Years	Numbers
**Central Africa**	Angola	2015–16	14,007
	Cameroon	2018	9,062
	DRC	2013–14	18,017
**West Africa**	Benin	2017–18	13,090
	Burkina-Faso	2010	14,592
	Ivory Coast	2011–12	7,413
	Gambia	2019–20	7,927
	Ghana	2014	5,642
	Guinea	2018	7,704
	Liberia	2019–20	5,490
	Mali	2018	9,748
	Mauritania	2019–21	11,110
	Nigeria	2018	33,016
	Senegal	2019	5,859
	Sierra Leone	2019	9,664
	Togo	2013–14	6,658
**East Africa**	Burundi	2017–18	12,989
	Comoros	2012	2,999
	Ethiopia	2016	10,408
	Madagascar	2021	12,265
	Malawi	2015–16	16,879
	Mozambique	2011	10,775
	Rwanda	2019–20	7,878
	Tanzania	2015–16	9,586
	Uganda	2016	14,808
	Zambia	2018	9,597
	Zimbabwe	2018	5,777
**Southern Africa**	Lesotho	2014	2,864
	Namibia	2013	4,809
	South Africa	2016	3,352
**SSA**		2010–21	303,985

### 2.4. Variables

#### 2.4.1. Dependent variables

The dependent variable was the status (1 = deceased vs. 0 = alive) of the child reported by the mother (or caregiver).

#### 2.4.2. Main exposures

These were the individual WASH conditions in which the children lived. These were assessed in the children’s immediate environment, i.e., in their households. The level of access to water, sanitation, and hygiene was grouped as “basic” (at least basic), “limited,” “unimproved,” and “no service” (Table 2). Additional information on standard definitions of these WASH service levels is available in the World Health Organization (WHO)/United Nations Children’s Fund (UNICEF) Joint Monitoring Program (JMP) thematic reports on progress in drinking water supply, sanitation, and hygiene (22).

**Table 2:** WHO/UNICEF JMP scale for WASH services.

Service ladder	Water	Sanitation	Hygiene
Basic	Improved water point^1^ providing drinking water, where the round trip to collect water does not exceed 30 min, including waiting time	Improved facility^2^ not shared with other households	Hand hygiene facility with soap and water available at home
Limited	Improved water point^1^ providing drinking water, where the round trip to collect water exceeds 30 min, including waiting time	Improved facility^2^ shared with other households	Handwashing facility without water or soap available at home
Unimproved	Water point from an unprotected well or unprotected spring	Flush and pour-flush toilets flushed to an open drain or elsewhere, pit latrines without slabs, open pits, hanging toilets/latrines, bucket latrines, including pans, trays or other unsealed containers	Not applicable
No service	Surface water: water directly from a river, dam, lake, pond, stream, canal or irrigation canal	Open defecation	No handwashing facility available in the home

Adapted from WHO; UNICEF. Progress on Drinking Water, Sanitation and Hygiene: 2017 Update and SDG Baselines; WHO: Geneva, 2017; ISBN 978–92-4-151,289-3.

1 Piped supplies (tap water in the dwelling, yard or plot, public stand posts) and non-piped supplies (boreholes/tubewells, protected wells and springs, rainwater, packaged water, including, bottled water and sachet water, delivered water, including tanker trucks and small carts).

2 Networked sanitation (flush and pour flush toilets connected to sewers) and on-site sanitation (flush and pour flush toilets or latrines connected to septic tanks or pits, ventilated improved pit latrines, pit latrines with slabs, composting toilets, including twin pit latrines and container-based systems).

#### 2.4.3. Other covariates

The other explanatory variables are related to the child, mother (or caregiver), household, and environment. The child variables included: number of months between birth and survey (≤11, 12–23, 24–35, 36–47, 48–59), sex (male, female), rank (1, 2, 3 and above), and type of birth (twin, single). Birth weight was not included due to missing data. Variables related to mothers of children were: age (15–20, 20–29, 30–39, 40–49), level of education (no formal education, primary, secondary, higher), marital status (in couple: married or live-in relationship, single), occupation (yes, no), and media exposure including newspapers, radio, and television (not at all, less than once a week, at least once a week). We did not include religion and health insurance coverage due to missing data. Household variables were: sex of the household head (male, female), wealth index (poorest, poorer, middle, richer, richest), and household size (≤5, >5). The environmental variables concerned: area (urban, rural), subregion (West Africa, East Africa, Central Africa, Southern Africa), and phase of the survey (Phase 6: 2008–2013, Phase 7: 2013–2018, Phase 8: 2018–2023).

### 2.5. Data analysis

All analyses took account of the sampling plans of the different surveys whose datasets were selected. The data analysis was carried out in two stages. One is descriptive, and the other is analytical. In the descriptive stage, the study variables were presented according to weighted numbers and percentages of their respective categories. In particular, the percentage of deceased children was calculated with the 95% Confidence Interval (95% CI). Also, the proportion of children covered by individual WASH services was determined according to the WHO/UNICEF JMP service scales.

The analytical step focused on identifying predictors of under-five mortality from which we inferred the effects of individual WASH conditions. We used mixed logistic regression (fixed and random effects) due to the hierarchical nature of the DHS data, which may prevent the assumptions of the “traditional” logistic regression: independence of observations and homoscedasticity (equality of variances). In line with other recent studies, we used two levels: children under five (level 1) and PSU (level 2) (23, 24). Identifying the effects of individual WASH conditions on under-five mortality, adjusted for covariates, was done in two phases: univariate and multivariate analysis. The univariate analysis aimed to cross the dependent variable with each covariate. The point is to determine which ones to take in the multivariate analysis. For this purpose, *p* < 0.20 was considered (25). For the multivariate analysis, we estimated a range of models using the individual WASH variables and the covariates selected from the univariate analysis. First, an empty model (Model 0) was regressed to decompose the total variance into inter-and intra-PSU variance. Model 1 introduced the individual WASH variables. Model 2 progressively introduced child, mother, household, and environment covariates (selected after the univariate analysis) in a step-by-step forward strategy (26). The significance level was 5%. We maintained the variables related to individual WASH conditions in the different models regardless of their significance. We calculated Odds Ratios (OR) and 95% CI to quantify fixed effects. Intraclass Correlation (ICC), Median Odds Ratio (MOR), and Proportional Change of Variance (PCV) served to measure random effects (27, 28). The ICC quantifies the heterogeneity in under-five mortality across PSUs (27, 28). The MOR is the median value of the range of odds ratios obtained by comparing mortality in children from two randomly selected distinct PSUs (27, 28). The PCV highlights the proportion of variability in under-five mortality explained by variables ultimately retained in Model 2. Random effects precision was based on the standard error (SE) of covariates and p. (29). We checked the fit of consecutive models using the Bayesian Deviance Information Criterion (DIC). The DIC decreases as fixed or random effects are added to the model; lower DIC values indicate a better model fit (30). We use Stata 15 (StataCorp, College Station, TX, United States) for data analysis.


$$ICC=\frac{\sigma^{2}}{\sigma^{2}+\frac{\pi^{2}}{3}}\;MOR=\exp^{\sqrt{2\sigma^{2}\times 0\mathrm{.6745}}}\;PCV=\frac{V_{0}-V_{2}}{V_{0}}$$

$$\sigma^{2}:inter-PSU\;variance$$

$$\frac{\pi^{2}}{3}\approx 3.29$$

$$V_{0}:varianceofModel\;0$$

$$V_{2}:varianceofModel\;2$$

## 3. Results

### 3.1. Description of the study population

Table 3 shows the basic characteristics of surveyed children under five. There were 303,985 (weighted number: 302,946) children in the study. They were predominantly male (50.71%) and had a birth rank of three or above (58.20%). About 2% of the children were twins. Most children’s mothers were between 20 and 39 years (85.03%), in a couple (87.10%), and had no formal education (38.70%). More than 70% of the mothers had a professional activity. Of the children’s mothers, 5.44, 33.76, and 23.13% read newspapers, listened to the radio, and watched television at least once a week, respectively. Most children lived in households headed by men (79.71%) and with more than five people (57.67%). We note that 23.11% of children were in households in the lowest quintile, while 16.04% were in households in the highest quintile. About seven out of 10 (68.76%) children lived in rural areas.

**Table 3:** Basic characteristics of surveyed children in SSA, 2010–21.

Variables	*n*	%
Number of months between birth and survey		
≤11	74,615	24.63
12–23	60,020	19.81
24–35	56,486	18.65
36–47	56,517	18.66
48–59	55,307	18.26
Child’s sex		
Male	153,617	50.71
Female	149,329	49.29
Child’s rank		
1	67,827	22.39
2	58,791	19.41
3 and above	176,328	58.20
Type of birth		
Single	296,229	97.78
Twin	6,716	2.22
Mother’s age		
15–19	18,028	5.95
20–29	148,737	49.10
30–39	108,873	35.94
40–49	27,308	9.01
Mother’s level of education		
No-formal education	117,236	38.70
Primary	103,634	34.21
Secondary	72,324	23.87
Higher	9,752	3.22
Mother’s marital status		
Single	39,094	12.90
In couple	263,852	87.10
Mother’s professional activity		
No	89,756	29.63
Yes	213,190	70.37
Mother’s exposure to newspapers		
Not at all	261,775	86.41
Less than once a week	24,697	8.15
At least once a week	16,474	5.44
Mother’s exposure to radio		
Not at all	137,191	45.29
Less than once a week	63,483	20.96
At least once a week	102,272	33.76
Mother’s exposure to television		
Not at all	193,864	63.99
Less than once a week	39,013	12.88
At least once a week	70,069	23.13
Household head’s sex		
Male	241,480	79.71
Female	61,466	20.29
Household wealth index		
Poorest	70,011	23.11
Poorer	66,260	21.87
Middle	61,069	20.16
Richer	57,001	18.82
Richest	48,606	16.04
Household size		
≤5	128,236	42.33
>5	174,710	57.67
Area		
Urban	94,621	31.23
Rural	208,325	68.77
Subregion		
Central	40,262	13.29
West	136,490	45.05
East	115,359	38.08
South	10,835	3.58
Phase		
Phase 6	65,221	21.53
Phase 7	175,706	58.00
Phase 8	62,018	20.47

### 3.2. Mortality of children under five

Overall, 6.36% (95% CI = 6.24–6.49) of children under five were deceased, with subregional disparities (Table 4). In Central Africa, the proportion of dead children before five was 6.29% (95% CI = 5.96–6.64), with 5.12% (95% CI = 4.58–5.71) in Angola, 6.09% (95% CI = 5.51–6.73) in Cameroon, and 7.27% (95% CI = 6.71–7.86) in DRC. In West Africa, 7.41% (95% CI = 7.21–7.62) of children died before five, with a minimum of 3.02% (95% CI = 2.51–3.63) in Senegal, and a maximum of 9.56% (95% CI = 9.07–10.07) in Nigeria. In East Africa, 5.30% (95% CI = 5.13–5.47) of children under five were deceased, with a minimum of 3.53% (95% CI = 3.09–4.02) in Rwanda and a maximum of 7.28% (95% CI = 6.70–7.91) in Mozambique. In Southern Africa, the percentage of dead children under five was 3.54% (95% CI = 2.74–4.56) in South Africa, 4.30% (95% CI = 3.69–5.01) in Namibia, and 6.82% (95% CI = 5.81–7.99) in Lesotho, for a subregional mean of 4.72% (95% CI = 4.26–5.23).

**Table 4:** Under-five mortality by country in Sub-Saharan Africa, 2010–21.

Subregions/Countries	Year	Total	Under-five mortality				
			*n*	%	95% CI		
Central Africa	2013–18	40,262	2,533	6.29	5.96	–	6.64
Angola	2015–16	13,083	670	5.12	4.58	–	5.71
Cameroon	2018	9,461	576	6.09	5.51	–	6.73
DRC	2013–14	17,718	1,287	7.27	6.71	–	7.86
West Africa	2010–21	136,490	10,121	7.41	7.21	–	7.62
Benin	2017–18	13,124	901	6.87	6.37	–	7.41
Burkina-Faso	2010	14,922	1,319	8.84	8.26	–	9.46
Ivory Coast	2011–12	7,147	582	8.14	7.26	–	9.11
Gambia	2019–20	7,223	326	4.52	3.99	–	5.11
Ghana	2014	5,428	251	4.63	4.00	–	5.36
Guinea	2018	7,647	657	8.59	7.75	–	9.51
Liberia	2019–20	5,062	376	7.43	6.27	–	8.78
Mali	2018	10,113	721	7.13	6.45	–	7.88
Mauritania	2019–21	11,177	390	3.49	3.11	–	3.91
Nigeria	2018	33,339	3,186	9.56	9.07	–	10.07
Senegal	2019	5,388	163	3.02	2.51	–	3.63
Sierra Leone	2019	9,556	854	8.93	8.20	–	9.72
Togo	2013–14	6,365	394	6.19	5.50	–	6.97
East Africa	2011–21	115,359	6,112	5.30	5.13	–	5.47
Burundi	2017–18	13,403	773	5.77	5.26	–	6.31
Comoros	2012	3,086	126	4.08	3.20	–	5.19
Ethiopia	2016	10,767	592	5.50	4.79	–	6.30
Madagascar	2021	12,110	698	5.76	5.26	–	6.31
Malawi	2015–16	16,975	809	4.77	4.36	–	5.21
Mozambique	2011	11,399	830	7.28	6.70	–	7.91
Rwanda	2019–20	8,096	286	3.53	3.09	–	4.02
Tanzania	2015–16	9,446	493	5.22	4.66	–	5.84
Uganda	2016	14,556	721	4.95	4.54	–	5.39
Zambia	2018	9,474	452	4.77	4.22	–	5.39
Zimbabwe	2018	6,047	333	5.50	4.83	–	6.26
Southern Africa	2013–16	10,835	512	4.72	4.26	–	5.23
Lesotho	2014	2,844	194	6.82	5.81	–	7.99
Namibia	2013	4,583	197	4.30	3.69	–	5.01
South Africa	2016	3,408	121	3.54	2.74	–	4.56
SSA	2010–21	302,946	19,277	6.36	6.24	–	6.49

### 3.3. Level of household access to WASH

#### 3.3.1. Water

**Figure 2:**
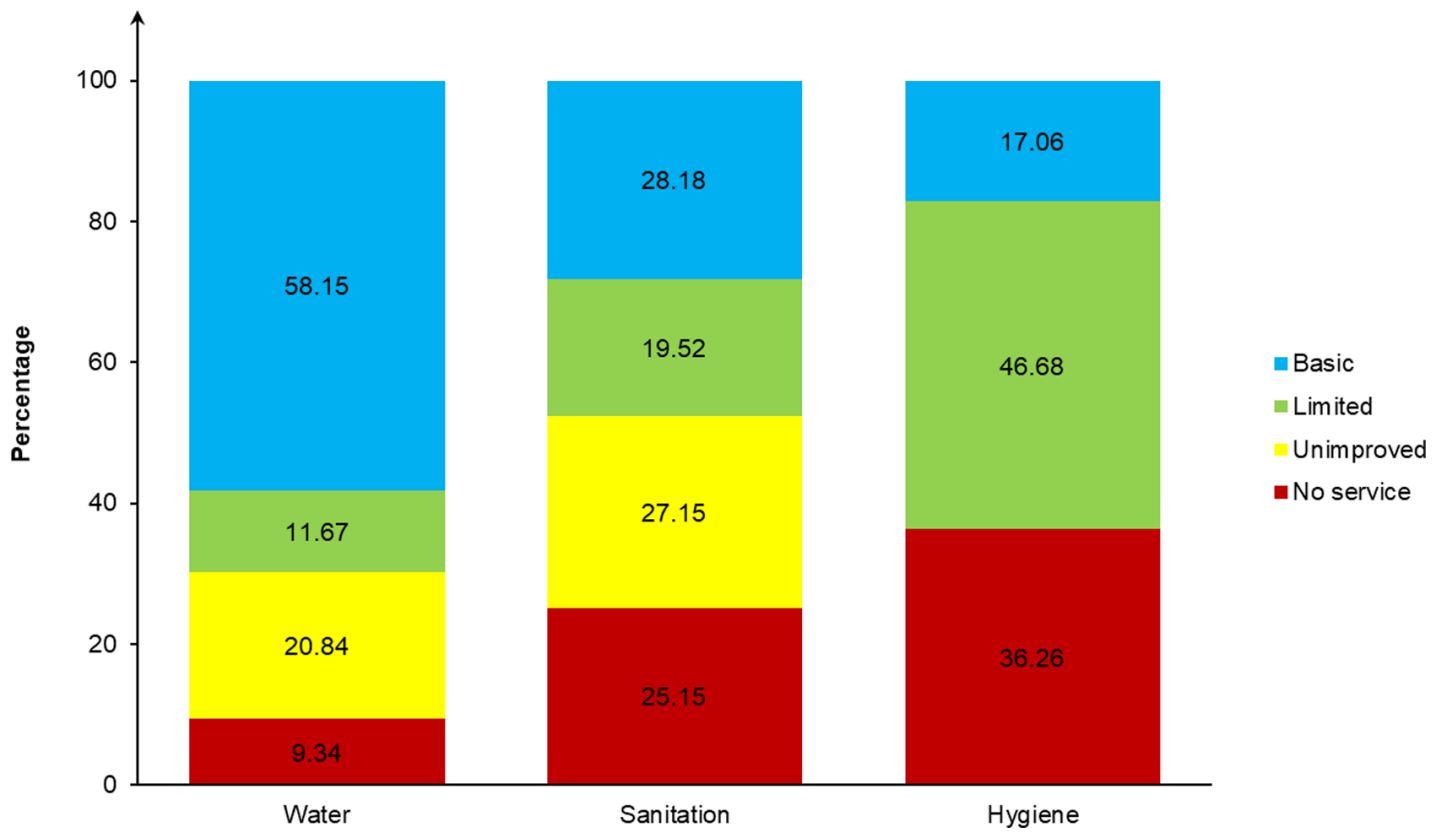
Distribution of children by access to household WASH services in SSA, 2010–21.

In SSA, the percentage of children living in households with access to basic water services was 58.15% (95% CI = 57.51–58.78; Figure 2), with subregional disparities. The distribution of children by household access to water services and subregion/country is provided in the Supplementary material.

In Central Africa, 47.35% (95% CI = 45.12–49.60) of children came from households with basic water facilities. DRC (33.90, 95% CI = 29.72–38.35) had the lowest coverage, while Cameroon (62.21, 95% CI = 58.77–65.53) had the highest. In West Africa, 64.85% (95% CI = 63.90–65.79) of children had access to basic water facilities, with a low of 53.03% (95% CI = 49.72–56.32) in Sierra Leone and a high of 89.68% (95% CI = 86.18–92.37) in Gambia. In Eastern Africa, just over half (52.00, 95% CI = 51.09–52.91) of the children had access to basic water services, with a low of 36.33% (95% CI = 33.42–39.34) in Madagascar and a high of 78.54% (95% CI = 74.04–82.45) in Comoros. In Southern Africa, Lesotho, Namibia, and South Africa had 71.84% (95% CI = 67.92–75.45), 76.56% (95% CI = 74.08–78.86), and 89.47% (95% CI = 87.08–91.46) of children living in households covered by basic water services, respectively, compared to a subregional mean of 79.38% (95% CI = 77.74–80.93). Another 9.34% (95% CI = 8.95–9.74) of children lived in households using surface water for drinking in SSA. This percentage was: 12.41% (95% CI = 11.03–13.93) in Central Africa, 7.66% (95% CI = 7.15–8.20) in West Africa, 10.82% (95% CI = 10.18–11.48) in East Africa, and 3.40% (95% CI = 2.67–4.33) in Southern Africa. According to these subregions, Angola (18.48, 95% CI = 16.15–21.06) in Central Africa, Sierra Leone (20.46, 95% CI = 17.81–23.39) in West Africa, Madagascar (26.46, 95% CI = 23.44–29.65) in East Africa and Namibia (5.23, 95% CI = 3.75–7.24) in Southern Africa were the top countries where children lived in households using surface water for drinking.

#### 3.3.2. Sanitation

In SSA, nearly two out of seven (28.18, 95% CI = 27.74–28.63) children lived in households with basic sanitation facilities. The distribution of children by household access to sanitation services and subregion/country is reported in the Supplementary material.

In Central Africa, 29.17% (95% CI = 27.46–30.93) of children lived in households with basic sanitation facilities. In this subregion, Angola, Cameroon, and DRC, 39.49% (95% CI = 36.90–42.14), 37.31% (95% CI = 33.99–40.76), and 17.19% (95% CI = 14.71–19.99) of children were covered by basic sanitation facilities, respectively. In West Africa, 24.99% (95% CI = 24.33–25.66) of children were in households with basic sanitation facilities. In this subregion, children in Benin (10.40, 95% CI = 9.17–11.77) and Togo (10.43, 95% CI = 9.09–11.94) were the least covered; in Senegal, they were the most covered, with more than half (54.70, 95% CI = 50.03–59.28) of the children living in households with basic sanitation facilities. In Eastern Africa, the coverage of children with basic sanitation ranged from 5.33% (95% CI = 4.44–6.39) in Ethiopia to 56.43% (95% CI = 54.62–58.23) in Rwanda, for a subregional coverage of 29.96% (95% CI = 29.37–30.55). In Southern Africa, 45.89% (95% CI = 43.89–47.90) of participants came from households with basic sanitation facilities, with a minimum of 26.75 (95% CI = 23.89–29.82) in Namibia and a maximum of 73.34% (95% CI = 69.99–76.44) in South Africa. Besides, slightly more than a quarter (25.15, 95% CI = 24.64–25.66) of the participants lived in households with no sanitation facilities (open defecation). This frequency was: 18.40% (95% CI = 17.08–19.80) in Central Africa, 32.84% (95% CI = 32.01–33.69) in West Africa, 17.73% (95% CI = 17.00–18.48) in East Africa, and 32.31% (95% CI = 30.49–34.18) in Southern Africa. According to the subregions, Angola (31.82, 95% CI = 29.27–34.48) in Central Africa, Burkina Faso (68.59, 95% CI = 66.39–70.71) in West Africa, Mozambique (44.13, 95% CI = 41.21–47.08) in East Africa, and Namibia (54.12, 95% CI = 50.87–57.33) in Southern Africa were the top countries where children lived in households without sanitation facilities (open defecation).

#### 3.3.3. Hygiene

In SSA, 17.06% (95% CI = 16.71–17.41) of children lived in households with basic hygiene facilities. The repartition of children by household access to hygiene services and subregion/country is provided in the Supplementary material. In Central Africa, 15.80% (95% CI = 14.76–16.89) of children were in households covered by basic hygiene facilities, with a minimum of 2.79% (95% CI = 2.21–3.51) in DRC and a maximum of 31.47% (95% CI = 29.34–33.68) in Cameroon. In West Africa, 16.81% (95% CI = 16.26–17.38) of children were covered by basic hygiene services, ranging from a low of 2.28% (95% CI = 1.52–3.41) in Liberia to a high of 36.18% (95% CI = 33.83–38.60) in Mauritania. In East Africa, 16.62% (95% CI = 16.14–17.12) of children came from households with basic hygiene facilities, with a minimum of 4.63% (95% CI = 3.98–5.38) in Burundi and a maximum of 46.51% (95% CI = 44.37–48.66) in Tanzania. In Southern Africa, 1.40% (95% CI = 0.78–2.50), 40.92% (95% CI = 38.42–43.47), and 37.72% (95% CI = 34.71–40.82) of children in Lesotho, Namibia, and South Africa, respectively, were in households with basic hygiene facilities.

### 3.4. Factors associated with under-five mortality

The full results of multivariate analysis by mixed logistic regression of factors associated with under-five mortality in SSA are in the Supplementary material.

#### 3.4.1. Random effects

In the empty model, the total variability in under-five mortality attributable to PSUs was 0.43 (95% CI = 0.40–0.46). According to the ICC, 11.46% (95% CI = 10.79–12.16) of the total variability in under-five mortality was due to variability between PSUs. In addition, the MOR was 1.86 (95% CI = 1.82–1.90): if two children were randomly selected from two different PSUs, the one living in the PSU with the highest mortality was 1.86 times more likely to die before the age of five than the other from the PSU with the lowest mortality. It indicates that there was significant heterogeneity in under-five mortality between the PSUs. In the final model, the total variability attributable to the PSUs (0.37, 95% CI = 0.34–0.40) remained significant but reduced; this shows that the variables retained in the final model explained 13.38% of the variance attributable to the PSUs observed in the empty model.

#### 3.4.2. Fixed effects

**Figure 3:**
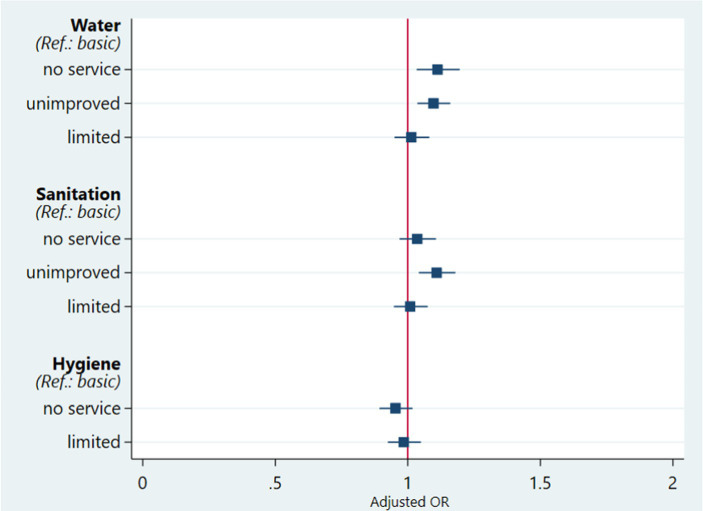
Effects of household access to WASH services by mixed logistic regression on under-five mortality, adjusted for covariates in SSA, 2010–21.

Children living in households using unimproved water facilities (aOR = 1.10; 95% CI = 1.04–1.16) or surface water for drinking (aOR = 1.11; 95% CI = 1.03–1.20) are more likely to die before five than those from households with basic water facilities (Figure 3). Children living in households with unimproved sanitation facilities have an 11% higher risk of under-five mortality (aOR = 1.11; 95% CI = 1.04–1.18) than those with basic sanitation facilities. We found no evidence to support a relationship between household access to hygiene services and under-five mortality.

Children born less than 12 months before the surveys were more likely to die (aOR = 115.36; 95% CI = 93.69–142.03) than those born 48–59 months ago. Male children had a 19% (aOR = 1.19; 95% CI = 1.14–1.24) higher risk of under-five mortality than females. Children with a birth rank of one (aOR = 1.86; 95% CI = 1.74–2.00) or two (aOR = 1.13; 95% CI = 1.06–1.20) were at greater risk of under-five mortality than those with a birth rank of three and above. Twins were 6.01 times (95% CI = 5.57–6.48) more likely to die before five than others. Children whose mothers were aged 20–49 years were from 2.05 (95% CI = 1.87–2.24) to 5.47 (95% CI = 4.86–6.15) times more likely to die before five than those whose mothers were aged 15–19 years. The risk of under-five mortality for children under five increased positively with mothers’ level of education. Compared to children of mothers with higher levels of education, the others had a 1.53 (95% CI = 1.31–1.80) to 2.29 (95% CI = 1.94–2.70) times higher risk of under-five mortality. Single and employed mothers were 1.41 (95% CI = 1.31–1.51) and 1.24 (95% CI = 1.18–1.30) times more likely to die before five than those whose mothers were married and unemployed, respectively. Children whose mothers frequently listened (at least once a week) to the radio (aOR = 1.08; 95% CI = 1.03–1.14) died more than those whose mothers did not. Also, the risk of under-five mortality was from 8% (95% CI = 1.01–1.15) to 9% (95% CI = 1.01–1.18) higher among children whose mothers watched television less than once a week than among those whose mothers were exposed at least once a week. Children in male-headed households were 21% more likely to under-five mortality (aOR = 1.21; 95% CI = 1.14–1.29) than children in female-headed households. Children living in households with five or fewer people (aOR = 1.83; 95% CI = 1.75–1.92) were at higher risk of under-five mortality than those from households with more than five people. Compared to children from the richest households, the others had from 18% (aOR = 1.18; 95% CI = 1.09–1.29) to 31% (aOR = 1.31; 95% CI = 1.19–1.44) higher risk of under-five mortality. Rural children had a 6% higher risk of under-five mortality (aOR = 1.06; 95% CI = 1.00–1.13) than those living in urban areas. The risk of under-five mortality was higher in Central Africa (aOR = 1.32; 95% CI = 1.23–1.42) and West Africa (aOR = 1.56; 95% CI = 1.48–1.64) compared to East Africa. There was a decrease in the risk of under-five mortality by the survey period, which was lower in phase 8 compared to phase 6 (aOR = 1.37; 95% CI = 1.29–1.47) and 7 (aOR = 1.29; 95% CI = 1.22–1.36).

## 4. Discussion

This work investigated the factors associated with under-five mortality using a large demographic and health dataset. As a result, we inferred the relative contribution of individual WASH conditions. We found that 6.36% of children born 5 years before the selected individual surveys had died. According to a 2019 study, the overall 15-year prevalence of under-five mortality in South Asian countries was 10% (31).

In the current study, children living in households using unimproved water facilities or surface water were more likely to die than those from households with basic water facilities. Surface water or water from an unimproved source is more likely to be contaminated with pathogens than water from a limited or basic source. Therefore, using water from unimproved sources to prepare weaning foods or to drink can promote the transmission to children of pathogens associated with diarrheal diseases, the fourth leading cause of death in children under five (32–34). Other studies have also found a significant relationship between the level of access to water and under-five mortality (9, 35). According to a 2020 study, increasing the quartile of the population’s access to an improved water source was associated with a decrease in the infant mortality rate of 1.14 deaths per 1,000 live births (35). A study based on 2010–2018 demographic and health data from 33 countries in SSA noted that the risk of under-five mortality was 19% higher among children from households using improved drinking water sources (9). However, in a multicenter study that combined Multiple Indicator Cluster Surveys (MICS) data from 41 low and middle-income countries, the authors did not find a significant relationship between household access to improved water sources and under-five mortality (14).

We found that children living in households with unimproved sanitation facilities have an 11% higher risk of under-five mortality than those with basic sanitation. In contrast to improved toilets, unimproved sanitation facilities do not guarantee that people will not come into contact with excreta. They lack water-based sanitation technologies (mechanical or manual flush toilets connected to the sewerage system, septic tanks, or pit latrines) and dry sanitation technologies (ventilated improved pit latrines, pit latrines with slab or composting toilets). Thus, the difficulties of cleaning, maintenance, and emptying associated with unimproved toilets limit the possibilities of preventing contamination and germ transmission between household members and between them and children. Also, in the first few months of life, children learning to crawl and walk or play may become exposed to pathogens from environmental sources through poor excreta disposal in unimproved sanitation facilities (36). Other studies have also found a significant relationship between the level of access to sanitation and under-five mortality (14, 35, 37). Increasing the quartile of the population’s access to improved toilets was linked to a decrease in the infant mortality rate of 1.66 deaths per 1,000 live births (35). MICS data from low and middle-income countries indicate that children from households with access to flush toilets had a 9–12% higher risk of under-five mortality than those from households without such facilities (14). A study in Asia suggested that the risk of under-five mortality was 49% higher for children living in households covered with unimproved facilities (37).

A literature review did not find any work on the influence of household access to hand hygiene facilities on under-five mortality. In the present study, we did not find a significant relationship between these two variables. Further studies are necessary to better understand and apprehend this result. It appears that the availability of water points with soap in households does not necessarily imply adherence to hand hygiene practices by household members at critical times such as after defecation or before eating or feeding children.

The study also found other factors associated with under-five mortality. Children born less than 12 months before the surveys were more likely to die than those born 48–59 months ago. According to the WHO, in 2019, children aged 0–11 months accounted for about 75% of the total number of deaths (5.2 million) observed in the under-fives worldwide (38). We found that males had a 19% higher risk of under-five mortality than females. This excess mortality in boys, which appears to be due to genetic and biological reasons, has also been recorded in other studies in Africa and Asia (5, 6, 8, 9, 31). In the present study, children with a birth rank of one or two were more likely to die than those with a birth rank of three and above. Unlike first-born children, those born later may benefit from the experience acquired by their parents in terms of child health and well-being as well as the support of their older siblings. A 2020 study recorded an inverse relationship (6). In the present work, twins were six times more likely to die than singles. Some complications (pre-eclampsia, eclampsia, gestational diabetes, etc.), known to increase maternal, neonatal, and infant mortality and morbidity, are more likely to occur in twin gestation (39, 40).

This study showed that children whose mothers were the oldest had an excess mortality risk compared to those with mothers in the youngest age group. Also, as the age of the mothers increased, the risk of under-five mortality increased. The results of this study corroborate the findings of other works (11, 14, 31). In contrast, a study in Asia found a negative relationship between under-five mortality and maternal age (37). According to the authors, older mothers are likely to be better prepared socially and mentally to care for their children (37). We observed a significantly higher mortality risk among children whose mothers were employed. A study in Bangladesh found a similar result (41). A possible explanation is insufficient attention to childcare related to work, with low compliance with exclusive breastfeeding (41). We found that compared to children of mothers with higher levels of education, the others had a 1.53 to 2.29 times higher risk of under-five mortality. A high level of education may play an important role in employment opportunities, the ability to make better decisions about health, increase in the resources required to ensure the physical and mental health of children. Children with single mothers were more likely to die than those with mothers in couples. Recent papers in DRC, Mali, Niger, and Zimbabwe found a similar result (11). Other studies suggest that maternal exposure to various media increases their ability to make decisions that benefit their health and that of their loved ones (42, 43). In the present study, the risk of under-five mortality was 8–9% lower in children whose mothers watched television compared to children of non-exposed mothers. Unexpectedly, maternal radio exposure was positively related to under-five mortality. Further studies will provide explanations for this relationship.

Under-five mortality was lower in wealthier households. This finding reflects the influence of economic status on under-five mortality, as shown in other studies (5, 6, 9, 11, 12, 14, 31). Households with favorable economic status are more likely to live in conditions that provide adequate housing, better nutrition and access to care for children, etc. (6). Mortality among children under the age of five being higher in households headed by men could be linked to their lower involvement in maternal and child care. The study also suggests excess under-five mortality in small households. In an Asian study, the authors found a lower risk of under-five mortality in larger households where more people are available and likely to care for the child (37). In addition, there is a disparity in under-five mortality by residence. Consistent with other studies, we found that urban areas were associated with lower mortality risk (5, 11, 12). A study noticed that this difference tends to disappear due to the increased access to health care and services in rural areas (31). In urban areas, many people become slum dwellers with poor health and living conditions (31). Besides, the risk of under-five mortality was higher in Central and West Africa than in East Africa.

The study had some strengths. The results of this study can be generalized to the population of children under five in the SSA countries studied during the period considered. The use of mixed logistic regression allowed for the hierarchical nature of the DHS data and variability within countries to provide reliable estimation and standard errors. We can point out some limitations of the present study. Given the cross-sectional nature of the data used in this study, a causal relationship between the exposures and the outcome variable cannot be established. We did not include 18 countries in the study for some reasons discussed above. Some variables were not studied because of missing data or reporting in a sub-sample (the last child). There is also a potential information bias in the status data of children. Furthermore, interactions between individual WASH facilities and target characteristics have not been explored.

## 5. Conclusion

Our analysis showed that a notable proportion of children under five in SSA lived in households without access to individual basic WASH services. We observed a negative and significant relationship between under-five mortality and access to water and sanitation services. Interventions to reduce under-five mortality should focus on improving access to basic water and sanitation services. We found no evidence to support a relationship between under-five mortality and access to hygiene services. Further studies are needed to investigate the influence of access to basic hygiene services on under-five mortality. Besides, differences related to the child, mother, and environmental characteristics were identified, revealing a profile of children who die before five. Children under 48 months (especially those under 12 months), male, first and second-born, twins; those with mothers who are older, poorly educated or uneducated, single, employed, listening to the radio, not watching television; as well as those living in households headed by men, poor, with less than five members, in rural areas, and in Central and West Africa, are at increased risk of mortality before their fifth birthday. In this context, we propose strengthening post-natal care for newborns, especially during the first 12 months and for twins; continuing efforts to improve basic childhood immunization and thereby prevent morbidity and mortality from vaccine-preventable diseases; enhancing family planning practices to space out births so that each child can receive the necessary attention and care to survive and thrive; providing information, education, and communication to older and low educated mothers on appropriate infant health care practices, including nutrition and exclusive breastfeeding; designing and implementing support systems for women raising their children alone; developing facilitators to enable the most deprived to subscribe to health insurance systems that match their economic situation; raising awareness to encourage men to take a more active role in child development; ongoing anti-poverty efforts; continuing to increase access to health services in rural areas. These approaches need to be implemented within an integrated and holistic approach to maximize their impact and ensure a reduction in child mortality.

## Data availability statement

The data used in this study can be obtained free of charge after a request via https://dhsprogram.com/.

## Ethics statement

The study used datasets from demographic and health surveys approved by ICF International’s internal ethics committee and national health research ethics authorities in Sub-Saharan Africa.

## Author contributions

All authors contributed to the conceptualization of the study. NG, AK, and CD collaborated on designing the methodology. NG conducted data acquisition and analysis and wrote the first draft of the manuscript. AK and CD supervised the data analysis. All authors revised the manuscript after the writing of the first version and after the peer review. All authors approved the final paper.

## Conflict of interest

The authors declare that the research was conducted in the absence of any commercial or financial relationships that could be construed as a potential conflict of interest.

## Publisher’s note

All claims expressed in this article are solely those of the authors and do not necessarily represent those of their affiliated organizations, or those of the publisher, the editors and the reviewers. Any product that may be evaluated in this article, or claim that may be made by its manufacturer, is not guaranteed or endorsed by the publisher.
