# Corrigendum: Effects of household access to water, sanitation, and hygiene services on under-five mortality in Sub-Saharan Africa

**DOI:** 10.3389/fpubh.2023.1280610

**Published:** 2023-09-28

**Authors:** Nicolas Gaffan, Alphonse Kpozehouen, Cyriaque Degbey, Yolaine Glele Ahanhanzo, Moussiliou Noël Paraïso

**Affiliations:** ^1^Department of Epidemiology and Biostatistics, Regional Institute of Public Health, University of Abomey-Calavi, Ouidah, Benin; ^2^Department of Environmental Health, Regional Institute of Public Health, University of Abomey Calavi, Ouidah, Benin; ^3^University Hospital Hygiene Clinic, National Hospital and University Centre Hubert Koutoukou Maga, Cotonou, Benin; ^4^Department of Health Promotion, Regional Institute of Public Health, University of Abomey-Calavi, Ouidah, Benin

**Keywords:** sanitation, hygiene, demographic and health survey, child, under-five mortality, household, Sub-Saharan Africa

In the published article, there was an error in the **Abstract**, *Results*. This sentence previously stated:

“The risk of under-five mortality was 11% higher for children living in households with limited sanitation facilities (aOR = 1.11; 95% CI = 1.04–1.18) than for those with basic sanitation services”.

The corrected sentence appears below:

“The risk of under-five mortality was 11% higher for children living in households with unimproved sanitation facilities (aOR = 1.11; 95% CI = 1.04–1.18) than for those with basic sanitation services”.

In the published article, there was an error in the **Results**, *Factors associated with under-five mortality, Fixed effects*, first paragraph. This sentence previously stated:

“Children living in households with limited sanitation facilities have an 11% higher risk of under-five mortality (aOR = 1.11; 95% CI = 1.04–1.18) than those with basic sanitation facilities”.

The corrected sentence appears below:

“Children living in households with unimproved sanitation facilities have an 11% higher risk of under-five mortality (aOR = 1.11; 95% CI = 1.04–1.18) than those with basic sanitation facilities”.

In the published article, there was an error in the **Discussion**, third paragraph. This sentence previously stated:

“We found that children living in households with limited sanitation facilities have a 9% higher risk of under-five mortality than those with basic sanitation”.

The corrected sentence appears below:

“We found that children living in households with unimproved sanitation facilities have an 11% higher risk of under-five mortality than those with basic sanitation”.

The authors apologize for these errors and state that this does not change the scientific conclusions of the article in any way. The original article has been updated.

