# Supplementary material for: Effects of household access to water, sanitation, and hygiene services on under-five mortality in Sub-Saharan Africa
Source: Front Public Health. 2023 Apr 27;11:1136299. doi: 10.3389/fpubh.2023.1136299 (PMC10173862; doi:10.3389/fpubh.2023.1136299)
Supplement: Supplementary file 1 [file Table_1.pdf]

Supplementary Material

Effects of Household Access to Water, Sanitation and Hygiene Services on Under-Five Mortality in Sub-Saharan Africa

Nicolas Gaffan[1*], Alphonse Kpozehouen[1], Cyriaque Degbey[2,3], Yolaine Glele Ahanhanzo[1], Moussiliou Noël Paraïso[4]

[1] Department of Epidemiology and Biostatistics, Regional Institute of Public Health, University of Abomey-Calavi, Ouidah, Benin

[2] Department of Environmental Health, Regional Institute of Public Health, University of Abomey Calavi, Ouidah, Benin

[3] University Hospital Hygiene Clinic, National Hospital and University Centre Hubert Koutoukou Maga, Cotonou, Benin

[4] Department of Health Promotion, Regional Institute of Public Health, University of Abomey Calavi, Ouidah, Benin

*** Correspondence:**
Nicolas Gaffan
gafnicolas@gmail.com

Notes

List of acronyms and abbreviations

95% CI	:	95% Confidence Interval
aOR	:	Adjusted Odds Ratios
cOR	:	Crude Odds Ratios
p	:	p-value

List of tables

Table S1. Distribution of children under five by household access to water in Sub-Saharan Africa, 2010-21 2

Table S2. Distribution of children under five by household access to sanitation in Sub-Saharan Africa, 2010-21......... 3

Table S3. Distribution of children under five by household access to hygiene in Sub-Saharan Africa, 2010-21 4

Table S4. Univariate analysis by mixed logistic regression of factors associated with under-five mortality in Sub-Saharan Africa, 2010-21 5

Table S5. Multivariate analysis by mixed logistic regression of factors associated with under-five mortality in Sub-Saharan Africa, 2010-21 7

Table S1. Distribution of children under five by household access to water in Sub-Saharan Africa, 2010-21

Subregions/ Countries	Years	Total	No service (surface water)			Unimproved			Limited			Basic		
			n	%	95% CI	n	%	95% CI	n	%	95% CI	n	%	95% CI
**Central Africa**	**2013-18**	**40,262**	4,996	12.41	11.03 - 13.93	11,790	29.28	27.11 - 31.55	4,410	10.95	9.85 - 12.17	19,066	47.35	45.12 - 49.60
Angola	2015-16	13,083	2,418	18.48	16.15 - 21.06	2,103	16.07	14.24 - 18.10	1,388	10.61	9.15 - 12.27	7,174	54.83	52.06 - 57.57
Cameroon	2018	9,461	850	8.99	6.37 - 12.54	1,924	20.34	17.81 - 23.12	801	8.47	7.33 - 9.77	5,885	62.21	58.77 - 65.53
DRC	2013-14	17,718	1,729	9.76	7.75 - 12.20	7,763	43.81	39.42 - 48.31	2,220	12.53	10.42 - 15.00	6,007	33.90	29.72 - 38.35
**West Africa**	**2010-21**	**136,490**	10,452	7.66	7.15 - 8.20	26,685	19.55	18.72 - 20.41	10,843	7.94	7.53 - 8.37	88,510	64.85	63.90 - 65.79
Benin	2017-18	13,124	928	7.07	5.63 - 8.83	3,381	25.76	23.38 - 28.30	1,130	8.61	7.49 - 9.88	7,685	58.56	55.92 - 61.14
Burkina-Faso	2010	14,922	1,084	7.26	5.93 - 8.87	2,792	18.71	16.68 - 20.93	1,722	11.54	10.22 - 13.01	9,324	62.49	60.03 - 64.88
Ivory Coast	2011-12	7,147	427	5.98	4.37 - 8.12	1,353	18.93	15.44 - 23.00	474	6.64	5.33 - 8.24	4,892	68.45	64.33 - 72.30
Gambia	2019-20	7,223	0	0.00		502	6.95	4.61 - 10.36	244	3.37	2.62 - 4.33	6,478	89.68	86.18 - 92.37
Ghana	2014	5,428	541	9.97	7.53 - 13.07	195	3.60	2.68 - 4.81	463	8.52	6.77 - 10.68	4,229	77.91	74.67 - 80.85
Guinea	2018	7,647	510	6.67	4.92 - 8.98	1,259	16.46	14.32 - 18.86	1,559	20.38	18.07 - 22.91	4,320	56.49	53.19 - 59.73
Liberia	2019-20	5,062	424	8.38	6.65 - 10.51	468	9.25	7.05 - 12.05	519	10.25	8.23 - 12.71	3,650	72.12	68.13 - 75.78
Mali	2018	10,113	192	1.90	1.26 - 2.86	3,152	31.17	27.40 - 35.20	380	3.76	2.83 - 4.98	6,388	63.17	59.37 - 66.81
Mauritania	2019-21	11,177	193	1.73	1.04 - 2.87	2,556	22.87	19.97 - 26.06	1,517	13.58	11.50 - 15.95	6,909	61.82	58.14 - 65.37
Nigeria	2018	33,339	3,123	9.37	8.08 - 10.83	7,166	21.49	19.40 - 23.75	1,190	3.57	2.97 - 4.29	21,861	65.57	63.22 - 67.85
Senegal	2019	5,388	0	0.00		874	16.22	13.04 - 20.00	296	5.49	3.34 - 8.91	4,218	78.28	72.99 - 82.79
Sierra Leone	2019	9,556	1,955	20.46	17.81 - 23.39	1,637	17.14	15.06 - 19.43	895	9.37	7.92 - 11.05	5,068	53.03	49.72 - 56.32
Togo	2013-14	6,365	1,075	16.90	13.68 - 20.68	1,348	21.18	17.96 - 24.79	454	7.13	5.82 - 8.69	3,488	54.80	51.15 - 58.41
**East Africa**	**2011-21**	**115,359**	12,478	10.82	10.18 - 11.48	23,579	20.44	19.67 - 21.23	19,315	16.74	16.15 - 17.35	59,987	52.00	51.09 - 52.91
Burundi	2017-18	13,403	638	4.76	3.60 - 6.28	1,764	13.16	11.59 - 14.92	2,726	20.34	18.30 - 22.54	8,274	61.73	59.00 - 64.40
Comoros	2012	3,086	48	1.54	0.53 - 4.37	300	9.71	7.07 - 13.19	315	10.21	7.98 - 12.98	2,424	78.54	74.04 - 82.45
Ethiopia	2016	10,767	1,352	12.56	10.18 - 15.38	3,279	30.46	26.48 - 34.75	1,712	15.90	13.20 - 19.03	4,424	41.09	37.21 - 45.08
Madagascar	2021	12,110	3,201	26.43	23.44 - 29.65	3,838	31.70	28.98 - 34.55	671	5.54	4.53 - 6.76	4,399	36.33	33.42 - 39.34
Malawi	2015-16	16,975	643	3.79	3.03 - 4.72	1,662	9.79	8.54 - 11.19	4,276	25.19	23.52 - 26.94	10,394	61.23	59.25 - 63.18
Mozambique	2011	11,399	1,886	16.55	14.22 - 19.16	3,885	34.08	31.23 - 37.04	1,248	10.95	9.38 - 12.75	4,380	38.43	35.52 - 41.42
Rwanda	2019-20	8,096	669	8.27	6.33 - 10.74	1,071	13.22	11.65 - 14.98	2,013	24.87	22.86 - 26.99	4,342	53.64	50.85 - 56.40
Tanzania	2015-16	9,446	1,346	14.25	11.84 - 17.05	2,659	28.14	25.12 - 31.38	1,208	12.78	10.98 - 14.83	4,234	44.82	41.79 - 47.89
Uganda	2016	14,556	1,314	9.03	7.57 - 10.73	1,965	13.50	11.91 - 15.27	3,726	25.60	23.96 - 27.31	7,551	51.87	49.47 - 54.27
Zambia	2018	9,474	840	8.87	7.37 - 10.63	2,213	23.36	20.93 - 25.98	714	7.54	6.31 - 8.98	5,707	60.24	57.27 - 63.13
Zimbabwe	2018	6,047	541	8.95	6.67 - 11.90	943	15.60	13.32 - 18.19	705	11.66	10.01 - 13.54	3,857	63.79	60.48 - 66.97
**Southern Africa**	**2013-16**	**10,835**	369	3.40	2.67 - 4.33	1,074	9.91	8.76 - 11.20	791	7.30	6.46 - 8.25	8,601	79.38	77.74 - 80.93
Lesotho	2014	2,844	19	0.66	0.33 - 1.30	497	17.47	14.34 - 21.12	285	10.03	8.34 - 12.01	2,043	71.84	67.92 - 75.45
Namibia	2013	4,583	240	5.23	3.75 - 7.24	441	9.62	8.03 - 11.48	394	8.60	7.10 - 10.38	3,509	76.56	74.08 - 78.86
South Africa	2016	3,408	110	3.23	2.27 - 4.59	137	4.01	3.00 - 5.33	112	3.29	2.49 - 4.35	3,049	89.47	87.08 - 91.46
**Sub-Saharan Africa**	**2010-21**	**302,946**	**28,295**	**9.34**	**8.95 - 9.74**	**63,128**	**20.84**	**20.27 - 21.42**	**35,359**	**11.67**	**11.34 - 12.01**	**176,164**	**58.15**	**57.51 - 58.78**

Table S2. Distribution of children under five by household access to sanitation in Sub-Saharan Africa, 2010-21

Subregions/ Countries	Years	Total	No service (open defecation)			Unimproved			Limited			Basic		
			n	%	95% CI	n	%	95% CI	n	%	95% CI	n	%	95% CI
**Central Africa**	**2013-18**	**40,262**	7,407	18.40	17.08 - 19.80	13,046	32.40	30.40 - 34.48	8,066	20.03	18.61 - 21.54	11,742	29.17	27.46 - 30.93
Angola	2015-16	13,083	4,162	31.82	29.27 - 34.48	875	6.69	5.79 - 7.71	2,879	22.00	19.83 - 24.34	5,167	39.49	36.90 - 42.14
Cameroon	2018	9,461	598	6.32	4.76 - 8.34	3,863	40.83	37.57 - 44.18	1,470	15.54	13.88 - 17.36	3,530	37.31	33.99 - 40.76
DRC	2013-14	17,718	2,647	14.94	12.89 - 17.25	8,308	46.89	43.05 - 50.77	3,717	20.98	18.40 - 23.82	3,045	17.19	14.71 - 19.99
**West Africa**	**2010-21**	**136,490**	44,829	32.84	32.01 - 33.69	28,344	20.77	20.10 - 21.45	29,210	21.40	20.82 - 22.00	34,107	24.99	24.33 - 25.66
Benin	2017-18	13,124	7,816	59.56	56.73 - 62.32	1,681	12.81	11.32 - 14.46	2,263	17.24	15.72 - 18.87	1,365	10.40	9.17 - 11.77
Burkina-Faso	2010	14,922	10,235	68.59	66.39 - 70.71	1,043	6.99	5.91 - 8.26	1,880	12.60	11.42 - 13.88	1,764	11.82	10.69 - 13.05
Ivory Coast	2011-12	7,147	2,794	39.09	34.82 - 43.55	1,350	18.89	16.33 - 21.75	1,826	25.56	22.83 - 28.49	1,176	16.46	14.27 - 18.92
Gambia	2019-20	7,223	81	1.12	0.72 - 1.71	2,454	33.97	30.80 - 37.30	1,184	16.39	14.29 - 18.73	3,505	48.52	45.41 - 51.64
Ghana	2014	5,428	1,349	24.85	21.57 - 28.46	484	8.92	7.36 - 10.77	2,989	55.08	51.61 - 58.49	605	11.15	9.55 - 12.99
Guinea	2018	7,647	1,106	14.46	12.28 - 16.96	2,901	37.93	34.74 - 41.23	1,895	24.77	22.39 - 27.32	1,746	22.83	20.76 - 25.04
Liberia	2019-20	5,062	1,942	38.37	34.29 - 42.62	836	16.51	13.92 - 19.48	1,315	25.98	22.93 - 29.27	969	19.14	16.40 - 22.22
Mali	2018	10,113	1,006	9.94	8.03 - 12.26	3,728	36.86	33.80 - 40.03	2,459	24.32	22.16 - 26.61	2,920	28.88	26.63 - 31.23
Mauritania	2019-21	11,177	4,456	39.87	36.89 - 42.93	789	7.06	5.79 - 8.58	1,266	11.33	10.01 - 12.79	4,665	41.74	39.04 - 44.49
Nigeria	2018	33,339	7,610	22.83	21.09 - 24.67	8,903	26.70	25.06 - 28.41	6,401	19.20	17.83 - 20.64	10,425	31.27	29.61 - 32.98
Senegal	2019	5,388	773	14.35	10.79 - 18.84	899	16.69	14.17 - 19.57	768	14.26	12.02 - 16.83	2,947	54.70	50.03 - 59.28
Sierra Leone	2019	9,556	2,023	21.17	19.09 - 23.40	2,736	28.63	26.26 - 31.13	3,441	36.01	33.68 - 38.40	1,356	14.19	12.91 - 15.58
Togo	2013-14	6,365	3,638	57.15	53.52 - 60.71	540	8.48	6.59 - 10.84	1,524	23.94	21.80 - 26.22	664	10.43	9.09 - 11.94
**East Africa**	**2011-21**	**115,359**	20,450	17.73	17.00 - 18.48	40,525	35.13	34.36 - 35.90	19,825	17.19	16.66 - 17.73	34,559	29.96	29.37 - 30.55
Burundi	2017-18	13,403	336	2.51	2.10 - 2.99	6,356	47.42	45.51 - 49.34	1,207	9.01	7.85 - 10.31	5,504	41.07	39.26 - 42.89
Comoros	2012	3,086	13	0.43	0.19 - 0.97	1,946	63.04	58.28 - 67.55	343	11.10	8.84 - 13.86	785	25.43	22.15 - 29.02
Ethiopia	2016	10,767	4,000	37.15	32.52 - 42.04	5,696	52.90	48.36 - 57.40	497	4.62	3.91 - 5.44	574	5.33	4.44 - 6.39
Madagascar	2021	12,110	4,612	38.08	35.20 - 41.06	4,110	33.94	31.51 - 36.46	2,306	19.05	17.17 - 21.08	1,081	8.93	7.89 - 10.09
Malawi	2015-16	16,975	1,129	6.65	5.99 - 7.38	1,966	11.58	10.61 - 12.63	5,335	31.43	29.85 - 33.06	8,545	50.34	48.70 - 51.98
Mozambique	2011	11,399	5,030	44.13	41.21 - 47.08	3,705	32.50	30.37 - 34.71	449	3.94	3.25 - 4.77	2,215	19.43	17.87 - 21.09
Rwanda	2019-20	8,096	246	3.04	2.56 - 3.61	2,082	25.72	24.17 - 27.33	1,199	14.81	13.22 - 16.56	4,569	56.43	54.62 - 58.23
Tanzania	2015-16	9,446	1,245	13.18	11.18 - 15.49	1,738	18.39	16.74 - 20.17	2,183	23.11	21.32 - 25.01	4,280	45.31	42.92 - 47.71
Uganda	2016	14,556	1,058	7.27	6.27 - 8.41	8,546	58.71	56.55 - 60.84	2,463	16.92	15.35 - 18.62	2,489	17.10	15.73 - 18.55
Zambia	2018	9,474	1,106	11.67	10.21 - 13.31	3,643	38.46	35.61 - 41.38	2,106	22.23	20.01 - 24.63	2,619	27.64	25.29 - 30.12
Zimbabwe	2018	6,047	1,674	27.68	24.53 - 31.07	738	12.21	10.35 - 14.35	1,736	28.70	26.25 - 31.28	1,899	31.41	28.75 - 34.19
**Southern Africa**	**2013-16**	**10,835**	3,501	32.31	30.49 - 34.18	337	3.11	2.51 - 3.85	2,025	18.69	17.19 - 20.29	4,973	45.89	43.89 - 47.90
Lesotho	2014	2,844	898	31.57	27.81 - 35.59	21	0.75	0.43 - 1.31	678	23.83	20.88 - 27.04	1,247	43.85	40.13 - 47.64
Namibia	2013	4,583	2,480	54.12	50.87 - 57.33	236	5.16	3.99 - 6.64	640	13.97	11.90 - 16.34	1226	26.75	23.89 - 29.82
South Africa	2016	3,408	123	3.60	2.62 - 4.91	79	2.31	1.45 - 3.67	707	20.75	17.98 - 23.82	2,500	73.34	69.99 - 76.44
**Sub-Saharan Africa**	**2010-21**	**302,946**	**76,187**	**25.15**	**24.64 - 25.66**	**82,252**	**27.15**	**26.64 - 27.66**	**59,126**	**19.52**	**19.13 - 19.91**	**85,381**	**28.18**	**27.74 - 28.63**

Table S3. Distribution of children under five by household access to hygiene in Sub-Saharan Africa, 2010-21

Subregions/ Countries	Years	Total	No service			Limited			Basic		
			n	%	95% CI	n	%	95% CI	n	%	95% CI
**Central Africa**	**2013-18**	**40,262**	23,938	59.46	57.59 - 61.30	9,964	24.75	23.33 - 26.22	6,360	15.80	14.76 - 16.89
Angola	2015-16	13,083	8286	63.34	61.03 - 65.59	1,909	14.59	13.14 - 16.17	2,888	22.07	20.02 - 24.27
Cameroon	2018	9,461	394	4.17	3.13 - 5.53	6,090	64.36	62.08 - 66.58	2,977	31.47	29.34 - 33.68
DRC	2013-14	17,718	15,257	86.11	83.50 - 88.37	1,966	11.10	9.12 - 13.44	494	2.79	2.21 - 3.51
**West Africa**	**2010-21**	**136,490**	50,278	36.84	36.04 - 37.65	63,264	46.35	45.55 - 47.15	22,947	16.81	16.26 - 17.38
Benin	2017-18	13,124	5,746	43.78	41.45 - 46.14	6,239	47.54	45.25 - 49.84	1,139	8.68	7.64 - 9.84
Burkina-Faso	2010	14,922	3,991	26.74	23.77 - 29.95	9,754	65.37	62.32 - 68.30	1,177	7.89	6.96 - 8.93
Ivory Coast	2011-12	7,147	3,514	49.17	45.60 - 52.76	2,805	39.24	36.25 - 42.33	828	11.58	9.86 - 13.56
Gambia	2019-20	7,223	615	8.51	6.73 - 10.71	6,107	84.54	81.97 - 86.81	502	6.94	5.63 - 8.55
Ghana	2014	5,428	2,704	49.81	45.84 - 53.79	1,744	32.13	28.85 - 35.59	980	18.06	15.96 - 20.36
Guinea	2018	7,647	2,421	31.65	29.20 - 34.22	3,909	51.12	48.43 - 53.80	1,317	17.23	15.45 - 19.17
Liberia	2019-20	5,062	4,058	80.17	77.51 - 82.59	888	17.55	15.26 - 20.09	115	2.28	1.52 - 3.41
Mali	2018	10,113	3,232	31.96	28.64 - 35.47	5,698	56.35	52.94 - 59.70	1,183	11.70	10.31 - 13.25
Mauritania	2019-21	11,177	2,940	26.30	23.49 - 29.32	4,193	37.52	35.15 - 39.95	4,044	36.18	33.83 - 38.60
Nigeria	2018	33,339	6,496	19.48	18.32 - 20.71	18,105	54.31	52.59 - 56.01	8,738	26.21	24.68 - 27.80
Senegal	2019	5,388	3,476	64.52	60.86 - 68.02	1,230	22.83	19.43 - 26.63	682	12.65	9.91 - 16.01
Sierra Leone	2019	9,556	5,640	59.02	55.96 - 62.01	2,161	22.61	20.28 - 25.12	1,755	18.37	16.31 - 20.63
Togo	2013-14	6,365	5,447	85.58	83.94 - 87.07	431	6.77	5.72 - 7.99	487	7.65	6.60 - 8.85
**East Africa**	**2011-21**	**115,359**	31,677	27.46	26.77 - 28.16	64,505	55.92	55.23 - 56.60	19,177	16.62	16.14 - 17.12
Burundi	2017-18	13,403	190	1.42	1.03 - 1.94	12,592	93.95	93.10- 94.70	620	4.63	3.98 - 5.38
Comoros	2012	3,086	1,528	49.50	44.67 - 54.34	1,092	35.40	31.19 - 39.84	466	15.10	12.26 - 18.46
Ethiopia	2016	10,767	5,102	47.38	43.89 - 50.90	5,038	46.79	43.51 - 50.10	627	5.82	4.91 - 6.89
Madagascar	2021	12,110	1,755	14.49	13.11 - 15.99	8,784	72.54	70.81 - 74.20	1,571	12.97	11.78 - 14.27
Malawi	2015-16	16,975	2,904	17.11	16.00 - 18.28	12,671	74.65	73.16 - 76.08	1,400	8.24	7.25 - 9.37
Mozambique	2011	11,399	6,302	55.29	52.92 - 57.63	3,964	34.77	32.72 - 36.88	1,133	9.94	8.82 - 11.19
Rwanda	2019-20	8,096	1,376	17.00	15.71 - 18.38	4,846	59.86	58.09 - 61.60	1,874	23.14	21.42 - 24.97
Tanzania	2015-16	9,446	1,531	16.21	14.68 - 17.86	3,522	37.29	35.30 - 39.31	4,393	46.51	44.37 - 48.66
Uganda	2016	14,556	6,183	42.48	40.21 - 44.78	4,970	34.14	32.45 - 35.88	3,403	23.38	21.68 - 25.17
Zambia	2018	9,474	4,670	49.29	46.70 - 51.89	3,204	33.82	31.21 - 36.53	1,600	16.89	14.88 - 19.11
Zimbabwe	2018	6,047	136	2.25	1.50 - 3.35	3,822	63.20	60.74 - 65.60	2,089	34.55	32.13 - 37.05
**Southern Africa**	**2013-16**	**10,835**	3,950	36.45	34.69 - 38.26	3,685	34.01	32.32 - 35.73	3,201	29.54	28.01 - 31.12
Lesotho	2014	2,844	2,723	95.75	93.95 - 97.03	81	2.85	1.79 - 4.51	40	1.40	0.78 - 2.50
Namibia	2013	4,583	703	15.34	13.33 - 17.60	2,005	43.74	40.87 - 46.65	1,875	40.92	38.42 - 43.47
South Africa	2016	3,408	524	15.37	12.87 - 18.24	1,599	46.91	44.01 - 49.84	1,286	37.72	34.71 - 40.82
**Sub-Saharan Africa**	**2010-21**	**302,946**	**109,843**	**36.26**	**35.71 - 36.81**	**141,419**	**46.68**	**46.17 - 47.19**	**51,684**	**17.06**	**16.71 - 17.41**

Table S4. Univariate analysis by mixed logistic regression of factors associated with under-five mortality in Sub-Saharan Africa, 2010-21

**Variables**	**cOR**	**95% CI**	**p**
**Water**			
No service	1.22	1.14 - 1.30	<0.001
Unimproved	1.20	1.14 - 1.26	<0.001
Limited	1.04	0.98 - 1.11	0.198
Basic	1.00		
**Sanitation**			
No service	1.40	1.32 - 1.47	<0.001
Unimproved	1.26	1.19 - 1.33	<0.001
Limited	1.13	1.07 - 1.20	<0.001
Basic	1.00		
**Hygiene**			
No service	1.15	1.08 - 1.22	<0.001
Limited	1.11	1.05 - 1.18	<0.001
Basic	1.00		
**Number of months between birth and survey**			
≤11	93.65	76.18 - 115.13	<0.001
12-23	16.91	13.68 - 20.90	<0.001
24-35	10.02	8.10 - 12.41	<0.001
36-47	3.91	3.11 - 4.90	<0.001
48-59	1.00		
**Child's sex**			
Male	1.22	1.17 - 1.27	<0.001
Female	1.00		
**Child's rank**			
1	1.04	0.99 - 1.09	0.095
2	0.86	0.81 - 0.90	<0.001
3 and above	1.00		
**Type of birth**			
No	1.00		
Yes	6.06	5.69 - 6.45	<0.001
**Mother's age**			
15-19	1.00		
20-29	0.82	0.76 - 0.89	<0.001
30-39	0.84	0.77 - 0.91	<0.001
40-49	1.07	0.98 - 1.17	0.129
**Mother's level of education**			
No-formal education	1.96	1.69 - 2.28	<0.001
Primary	1.67	1.44 - 1.94	<0.001
Secondary	1.35	1.16 - 1.57	<0.001
Higher	1.00		

Table S4. continued

**Variables**	**cOR**	**95% CI**	**p**
**Mother's marital status**			
Single	1.06	1.00 - 1.12	0.050
In couple	1.00		
**Mother's professional activity**			
No	1.00		
Yes	1.11	1.06 - 1.16	<0.001
**Mother's exposure to newspapers**			
Not at all	1.40	1.26 - 1.55	<0.001
Less than once a week	1.12	0.99 - 1.27	0.076
At least once a week	1.00		
**Mother's exposure to radio**			
Not at all	1.03	0.99 - 1.08	0.156
Less than once a week	1.01	0.95 - 1.06	0.777
At least once a week	1.00		
**Mother's exposure to television**			
Not at all	1.30	1.23 - 1.37	<0.001
Less than once a week	1.23	1.14 - 1.32	<0.001
At least once a week	1.00		
**Household head's sex**			
Male	1.09	1.04 - 1.14	0.001
Female	1.00		
**Household wealth index**			
Poorest	1.62	1.52 - 1.74	<0.001
Poorer	1.60	1.50 - 1.72	<0.001
Middle	1.41	1.32 - 1.51	<0.001
Richer	1.32	1.22 - 1.42	<0.001
Richest	1.00		
**Household size**			
≤5	1.47	1.42 - 1.53	<0.001
>5	1.00		
**Area**			
Urban	1.00		
Rural	1.30	1.25 - 1.36	<0.001
**Subregion**			
Central	1.22	1.15 - 1.30	<0.001
West	1.42	1.36 - 1.47	<0.001
South	0.89	0.80 - 0.99	0.036
East	1.00		
**Phase**			
Phase 6	1.41	1.33 - 1.49	<0.001
Phase 7	1.21	1.15 - 1.27	<0.001
Phase 8	1.00		

Table S5. Multivariate analysis by mixed logistic regression of factors associated with under-five mortality in Sub-Saharan Africa, 2010-21

Variables	Model 0			Model 1			Model 2		
	aOR	95% CI	p	aOR	95% CI	p	aOR	95% CI	p
**RANDOM EFFECTS**									
**Community variance**	0.43	0.40 - 0.46		0.40	0.37 - 0.43		0.37	0.34 - 0.40	
**ICC**	11.46	10.79 - 12.16		10.81	10.15 - 11.50		10.08	9.36 - 10.86	
**MOR**	1.86	1.82 - 1.90		1.82	1.78 - 1.86		1.78	1.74 - 1.82	
**PCV**				6.40	6.13 - 6.67		13.38	12.06 - 14.68	
**FIXED EFFECTS**									
**Water**									
No service				1.14	1.06 - 1.22	<0.001	1.11	1.03 - 1.20	0.004
Unimproved				1.13	1.08 - 1.19	<0.001	1.10	1.04 - 1.16	0.001
Limited				1.02	0.96 - 1.08	0.631	1.01	0.95 - 1.08	0.689
Basic				1.00			1.00		
**Sanitation**									
No service				1.34	1.27 - 1.42	<0.001	1.04	0.97 - 1.11	0.303
Unimproved				1.22	1.16 - 1.29	<0.001	1.11	1.04 - 1.18	0.001
Limited				1.13	1.06 - 1.20	<0.001	1.01	0.95 - 1.07	0.775
Basic				1.00			1.00		
**Hygiene**									
No service				1.07	1.00 - 1.13	0.039	0.95	0.89 - 1.02	0.155
Limited				1.06	0.99 - 1.12	0.073	0.98	0.92 - 1.05	0.638
Basic				1.00			1.00		
**Number of months between birth and survey**									
≤11							115.36	93.69 - 142.03	<0.001
12-23							19.78	15.99 - 24.48	<0.001
24-35							11.08	8.95 - 13.73	<0.001
36-47							4.10	3.27 - 5.15	<0.001
48-59							1.00		
**Child's sex**									
Male							1.19	1.14 - 1.24	<0.001
Female							1.00		
**Child's rank**									
1							1.86	1.74 - 2.00	<0.001
2							1.13	1.06 - 1.20	<0.001
3 and above							1.00		
**Type of birth**									
No							1.00		
Yes							6.01	5.57 - 6.48	<0.001
**Mother's age**									
15-19							1.00		
20-29							2.05	1.87 - 2.24	<0.001
30-39							3.17	2.86 - 3.52	<0.001
40-49							5.47	4.86 - 6.15	<0.001
**Mother's level of education**									
No-formal education							2.29	1.94 - 2.70	<0.001
Primary							2.10	1.78 - 2.48	<0.001
Secondary							1.53	1.31 - 1.80	<0.001
Higher							1.00		

Table S5. Continued

Variables	Model 0			Model 1			Model 2		
	aOR	95% CI	p	aOR	95% CI	p	aOR	95% CI	p
**FIXED EFFECTS**									
**Mother's marital status**									
Single							1.41	1.31 - 1.51	<0.001
In couple							1.00		
**Mother's professional activity**									
No							1.00		
Yes							1.24	1.18 - 1.30	<0.001
**Mother's exposure to newspapers**									
Not at all									
Less than once a week									
At least once a week									
**Mother's exposure to radio**									
Not at all							1.00		
Less than once a week							1.02	0.96 - 1.08	0.594
At least once a week							1.08	1.03 - 1.14	0.002
**Mother's exposure to television**									
Not at all							1.08	1.01 - 1.15	0.032
Less than once a week							1.09	1.01 - 1.18	0.030
At least once a week							1.00		
**Household head's sex**									
Male							1.21	1.14 - 1.29	<0.001
Female							1.00		
**Household wealth index**									
Poorest							1.29	1.16 - 1.42	<0.001
Poorer							1.31	1.19 - 1.44	<0.001
Middle							1.21	1.10 - 1.32	<0.001
Richer							1.18	1.09 - 1.29	<0.001
Richest							1.00		
**Household size**									
≤5							1.83	1.75 - 1.92	<0.001
>5							1.00		
**Area**									
Urban							1.00		
Rural							1.06	1.00 - 1.13	0.039
**Subregion**									
Central							1.32	1.23 - 1.42	<0.001
West							1.56	1.48 - 1.64	<0.001
South							1.07	0.94 - 1.21	0.289
East							1.00		
**Phase**									
Phase 6							1.37	1.29 - 1.47	<0.001
Phase 7							1.29	1.22 - 1.36	<0.001
Phase 8							1.00		
